# Molecular Genetic and Functional Analysis of *pks*-Harboring, Extra-Intestinal Pathogenic *Escherichia coli* From India

**DOI:** 10.3389/fmicb.2018.02631

**Published:** 2018-11-15

**Authors:** Arya Suresh, Amit Ranjan, Savita Jadhav, Arif Hussain, Sabiha Shaik, Munirul Alam, Ramani Baddam, Lothar H. Wieler, Niyaz Ahmed

**Affiliations:** ^1^Pathogen Biology Laboratory, Department of Biotechnology and Bioinformatics, University of Hyderabad, Hyderabad, India; ^2^Department of Microbiology, Dr. D. Y. Patil Medical College, Hospital and Research Centre (Dr. D. Y. Patil Vidyapeeth), Pune, India; ^3^International Centre for Diarrhoeal Disease Research, Bangladesh (icddr,b), Dhaka, Bangladesh; ^4^Robert Koch Institute, Berlin, Germany

**Keywords:** genotoxins, *pks* island, colibactin, extraintestinal pathogenic *E. coli* (ExPEC), virulence

## Abstract

Colibactin, a genotoxin, encoded by the *pks* pathogenicity island of *Escherichia coli* belonging to the B2 phylogroup has been reported as a determinant of bacterial pathogenicity. The present study was carried out to detect the *pks* pathogenicity island in extraintestinal pathogenic *E. coli* (ExPEC) isolated from a tertiary hospital in Pune, India. Of 462 isolates analyzed, the *pks* genomic island was detected in 35 (7.6%) isolates, which predominantly belonged to pathogenic phylogroup B2 (97%), and harbored virulence genes such as *fimH, sfaD/E*, and *usp*. Biofilm formation assay revealed 21 of the 35 *pks-*carrying isolates to be strong (SBF > 1.0), 10 isolates to be moderate (SBF = 0.5–1.0), and 4 as weak (SBF < 0.5) biofilm formers. All of the *pks-*carrying isolates proved resistant against bactericidal activity of human serum. Assays carried out to detect antimicrobial susceptibility revealed 11% of these isolates to be multidrug resistant, 37% producing ESBL and 25% were positive for *bla*_CTX-M-15_. The observed prevalence of multidrug resistance and colibactin producing characteristics among pathogenic *E. coli* belonging to phylogenetic group B2 advocate urgent need for broader surveillance in order to understand and prevent transmission of these ExPEC in community and hospital settings.

## Introduction

Extraintestinal pathogenic *Escherichia coli* (ExPEC), apart from being a seasoned nosocomial pathogen is also an important cause of community-acquired (Johnson and Russo, 2002) and other infections such as urinary tract infection, sepsis, neonatal meningitis, and colibacillosis in humans and animals (Kaper et al., 2004; Smith et al., 2007). Genome analysis revealed that pathogenic *E. coli* have genome sizes in excess of about 1.0 Mb than the commensal strains mainly due to the presence of genes encoding multiple virulence factors, such as adhesins, toxins, invasins and siderophores that are absent or unlikely to be present in commensal strains (Croxen and Finlay, 2010). The virulence factors are mostly associated with phages and pathogenicity islands and undergo horizontal gene transfer which disseminates traits, thereby offering fitness advantages to recipient organisms (Ahmed et al., 2008).

Secreted toxins are the virulence factors that play an important role in long term colonization and pathogenesis of ExPEC. Some of these known secreted toxins comprise of important genotoxins, such as cytolethal distending toxins (CDT’s), cycle inhibiting factors (*cif’*s) and cytotoxic necrotizing factors (CNF’s) which can directly regulate the cell cycle of the host (Nougayrède et al., 2005). Colibactin is another important genotoxin produced by a 54-kb pathogenicity island known as *pks* island harbored by the members of *Enterobacteriaceae* (Nougayrède et al., 2006). This genomic island consists of a *clbA-S* gene cluster that encodes non-ribosomal peptides and polyketide synthases along with accessory and tailoring enzymes (Nougayrède et al., 2006). Colibactin acts as a cyclomodulin and blocks the eukaryotic cell cycle causing progressive enlargement of the nucleus as well as the cell body eventually leading to cell death (Cuevas-Ramos et al., 2010). The cytopathic effect of these genotoxins is mediated by live bacteria and requires a direct contact with the host cell (Nougayrède et al., 2006).

The *pks* island was first identified in sequenced genomes of ExPEC prototype strains and was detected predominantly in strains of phylogenetic group B2 (Johnson et al., 2008). The island was shown to display several signatures reminiscent of its horizontal acquisition (Putze et al., 2009; Messerer et al., 2017). The origin and prevalence of the colibactin island among enteric pathogens is largely unexplored. However, it was also found to be present in other members of *Enterobacteriaceae* such as *Citrobacter koseri, Klebsiella pneumoniae*, and *Enterobacter aerogenes* (Putze et al., 2009). Epidemiological studies demonstrated the prevalence of *pks* in ExPEC and their association with severe infections in different host populations of varied geographical locations (Johnson et al., 2008; Buc et al., 2013; Shimpoh et al., 2017).

Epidemiological studies on ExPEC in India have so far been focused on understanding antimicrobial resistance, evolution and presence of pandemic clones (Avasthi et al., 2011; Jadhav et al., 2011; Hussain et al., 2012, 2014; Shaik et al., 2017). However, a better understanding of ExPEC associated virulence factors may help in the development of therapeutic interventions, such as diagnostics and/or vaccines against ExPEC infections, and this would also facilitate risk assessment of ExPEC strains.

While colibactin is regarded as a virulence factor in ExPEC, not much is known in the context of its molecular epidemiology entailing Indian clinical isolates. Therefore, the present study was performed in order to investigate the prevalence and carriage of *pks* island, and to decipher the genotypic and functional characteristics of *pks* harboring clinical ExPEC isolates from India.

## Materials and Methods

### Bacterial Isolates

A total of 462 isolates of ExPEC were harnessed for this study. These isolates were originally collected by SJ and her colleagues from Dr D. Y. Patil University Hospital, Pune, India as a part of their routine diagnostic screening during the years 2009–2015 and were also described in our previous study (Ranjan et al., 2016). The bacterial collection of our study, essentially a sub-set of the collection studied by Ranjan et al. (2016), comprised 370 isolates cultured from urine, 63 from pus and 29 from other extra intestinal clinical samples. Standard microbiological laboratory methods were employed for the identification and preservation of these isolates (Jadhav et al., 2011). All isolates were collected, preserved and handled as per standard biosafety guidelines and according to the approvals of the Institutional Biosafety Committee (IBSC) of the University of Hyderabad (Ref. UH/IBSC/NA/12/7 dated 09/4/2012 and NA-N-32 dated 27/8/2015). The clinical information of the isolates is described in the Supplementary Table S1.

### Detection of *pks*-Genomic Island and Phylogroup Determination

The clinical isolates were screened for the presence of *pks* island by PCR using primers for the four representative genes of the genomic island so as to generate two flanking (*clbB* and *clbQ*) and two internal (*clbA* and *clbN*) amplicons in order to document presence of a complete island (Johnson et al., 2008). Heat killed bacterial lysates were used as DNA templates for PCR amplification, as described previously (Ranjan et al., 2017). PCR amplifications were carried out in 30 cycles at specific reaction conditions as described earlier (Johnson et al., 2008; Ranjan et al., 2017). The *pks*-positive isolates were assigned to one of the eight phylogroups based on multiplex-PCR amplification of four genes (*chuA, yjaA, arpA*, and *TspE4.C2*) as described elsewhere (Clermont et al., 2013).

### Antibiotic Susceptibility and Extended Spectrum-β-Lactamase Production

Antibiotic susceptibility analysis was performed, as previously described, by Kirby-Bauer disk diffusion method, on Mueller Hinton agar plates (Qumar et al., 2017). Antimicrobial disks (Himedia, India) specific for fosfomycin (200 μg), chloramphenicol (30 μg), co-trimoxazole (20 μg), tetracycline (30 μg), gentamicin (10 μg), nalidixic acid (30 μg), doxycycline (30 μg), ciprofloxacin (5 μg), and colistin (10 μg) were used to determine the antibiotic susceptibility profile of the isolates. ESBL production was determined using disk synergy between clavulanic acid and indicator cephalosporins, CTX (cefotaxime) and CAZ (ceftazidime). Both the assays were performed in accordance with Clinical Laboratory Standards Institute (CLSI) guidelines (CLSI, 2013). Isolates exhibiting resistance to three or more antimicrobials were designated as multidrug resistant (MDR).

### Virulence and Antimicrobial Resistance Genotyping

PCR based screening of virulence genes encoding bacterial adhesins (*fimH, sfaD/E, afa)*, toxins (*usp, cvaC, sat)*, iron acquisition system (*iucD)* and protectants (*ibeA)* were performed using primers and reaction conditions as previously described (Jadhav et al., 2011; Ranjan et al., 2017). ESBL gene *bla*_CTX-M-15_, (Monstein et al., 2007), genes conferring resistance to tetracycline (*tetA)*, sulfonamides (*sul1*) and aminoglycoside acetyl transferases (*aac(6*^′^)*-Ib)* were also screened for using primers and PCR conditions as described in previous studies (Jadhav et al., 2011; Ranjan et al., 2016, 2017). The isolates were also screened by PCR using generic primers for TEM and amplified products were sequenced to identify the variants of the gene.

### Determination of Siderophore Production, Biofilm Formation and Serum Resistance Assay

All the *pks-*positive isolates were screened for siderophore production using Chrome Azurol S Blue agar plates. A single colony of the bacterial isolate(s) was streaked on these plates and incubated overnight at 37°C. Colonies showing characteristic orange halos were identified as positive for siderophore production (Schwyn and Neilands, 1987).

All the 35 *pks-*positive isolates were analyzed for their biofilm forming capabilities as described previously (Nandanwar et al., 2014). Briefly, OD at 600 nm was taken for overnight grown bacterial cultures and all the isolates were diluted to an OD of 0.05 in fresh M63 minimal medium. An aliquot of 200 μL of the diluted culture was pipetted into flat-bottom 96 well sterile microtiter plates in triplicates. The plates were covered by a breathable sealing after obtaining OD at 600 nm [OD_600(0__h)_]. The plates were incubated for 48 h at stationary condition at 28°C. Following this, OD was obtained at 600 nm [OD_600(48__h)_]. Media was aspirated and wells washed thrice with 300 μL of deionized water. After air drying, bacteria were fixed using 250 μL of 99% methanol for 15 min and stained using 0.1% crystal violet solution for 30 min. Following staining, wells were washed thrice with deionized water and air dried. To solubilize the stained bacteria, 300 μL of Ethanol: Acetone (80:20) solution was added and incubated for 30 min at 100 rpm. OD at 570 nm was read in microtiter plate reader and specific biofilm formation was obtained using the following formula: SBF (specific biofilm formation) = (AB-CW)/G where AB = OD at 570 nm of attached and stained bacteria, CW = OD of control at 570 nm and G = OD_600(48__h)_ - OD_600(0__h)_, representing bacterial growth. The experiment was repeated twice in technical triplicates.

Serum resistance was also determined for all the 35 *pks*-positive *E. coli* isolates *in vitro* using 50% human serum as described earlier (Hussain et al., 2014). Briefly, 5 μL of overnight culture was added to 495 μl of LB broth and incubated in a shaking incubator at 37°C for 1 h at 200 rpm. The bacterial cultures were pelleted and resuspended in 1mL of 1X PBS; 30 μL of this inoculum was added to 270 μL of 50% human serum in triplicates in a 96 well microtiter plate. In each case, an initial sample was collected and plated after dilution on LB agar plates for enumerating the colony forming units (CFU) at 0 h. The inoculated plate was incubated for 3 h at 37°C at 100 rpm. After 3 h, samples from each well were serially diluted and plated on LB agar plates for 3 h count. Isolates which had equal or higher CFU counts at 3 h compared to 0 h were considered resistant to human serum. Growth was obtained by subtracting CFU counts of 0 h from that of 3 h. The experiment was repeated two times in technical triplicate(s).

### Statistical Analysis

All statistical calculations were performed using GraphPad Prism (version 5.01). Non-parametric Mann–Whitney *U* test was performed for serum resistance assay. *p*-values ≤ 0.05 were considered to be significant and were denoted in the graph.

## Results

### Screening for *pks* Island and Phylogenetic Grouping

A total of 462 *E. coli* isolates from our collection were screened for the presence of *pks* island, of which 35 were found to be positive for all the four targeted genes (*clbA, clbB, clbN* and *clbQ)* amplified from flanking and internal regions. Of these, 30 were originally cultured from urine, four from pus and one from blood. The prevalence of *pks*-positive isolates was 7.6% of the total *E. coli* collection studied. Using multiplex PCR, identification of *E. coli* phylogenetic groups was performed and out of the 35 isolates, 34 belonged to phylogroup B2 while one was assigned to phylogroup D (Table 1).

**Table 1:** Phylogroups, virulence and resistance genotypes, and antimicrobial resistance of *pks*-positive *E. coli* isolates.

Genotypic characterization		No. (%) of positive isolates
**Phylogenetic group**		
B2		34 (97.14)
D		1 (2.86)
**Virulence factors: Genotypic determinant**		
Adhesins	*fimH*	35 (100)
	*sfaD/E*	35 (100)
	*afa*	0 (0)
Toxins	*usp*	35 (100)
	*sat*	12 (34.29)
	*cvaC*	15 (42.86)
Protectins	*ibeA*	11 (31.43)
Iron acquisition	*iucD*	18 (51.43)
**Resistance factors: Antibiotic class**		
Tetracyclines	*tetA*	2 (5.71)
Fluoroquinolones	*aac(6*^′^*)-Ib*	7 (20)
Sulfonamides	*sul1*	4 (11.43)
β-lactamases	*bla*_TEM-1_	8 (22.86)
	*bla*_CTX-M-15_	9 (25.71)

**Antimicrobial class or phenotype**	**Specific Drug**	**No. (%) of resistant isolates**

Aminoglycoside	Gentamicin	2 (5.71)
Tetracyclines	Tetracycline	8 (22.86)
	Doxycycline	4 (11.43)
Sulfonamide/trimethoprim	Co-trimoxazole	5 (14.29)
Phenicol	Chloramphenicol	0 (0)
Phosphonic acid derivative	Fosfomycin	0 (0)
Fluoroquinolone	Ciprofloxacin	2 (5.71)
	Nalidixic Acid	25 (71.43)
Antibacterial peptide	Colistin	0 (0)
Multidrug Resistance		4 (11.42)
ESBL		13 (37.14)

### Antimicrobial Susceptibility and ESBL Production

Antimicrobial susceptibility testing against nine different antimicrobial agents belonging to seven different antibiotic classes revealed that the isolates were only moderately resistant to antibiotics. Maximum resistance was observed against nalidixic acid (71.4%). The isolates were found to be less resistant to tetracycline (22.86%), co-trimoxazole (14.29%), doxycycline (11.43%), gentamicin (5.71%), and ciprofloxacin (5.71%) and were completely sensitive to fosfomycin, chloramphenicol, and colistin. Of the 35 *E. coli* isolates, 13 (37.14%) were found to be ESBL producers and 4 (11.42%) were multidrug resistant. The details of resistance profile(s) for each antibiotic tested are shown in Table 1.

### Virulence and Resistance Genotyping

PCR based virulence and resistance genotyping of *pks*-positive isolates revealed their being relatively virulent as they possessed higher number of virulence genes compared to the resistance genes detected. Among bacterial adhesins tested, *fimH* and *sfaD/E* were 100% prevalent while *afa* was not detected in any of the isolates. Screening of toxin genes revealed presence of *usp* gene among all the isolates (100%), whereas *cvaC* and *sat* showed 42.86% and 34.29% prevalence, respectively. The genes *iucD* (iron acquisition system) and *ibeA* (protectant) were present in 51.43% and 31.43% of the isolates, respectively (Table 1).

Antimicrobial resistance genotyping revealed that the ESBL gene *bla*_CTX-M-15_ was present in 25.71% (*n* = 9) of the isolates and all these isolates were phenotypically observed to be ESBL producers by double disk synergy test. Further, 22.86% (*n* = 8) of the isolates were positive for TEM, and sequencing of PCR products followed by BLAST analysis identified all the amplicons to be entailing *bla*_TEM-1_. The gene *aac(6*^′^*)-Ib*, which is involved in aminoglycoside resistance, was present in 20% of the *pks*-positive isolates. Occurrence of genes that confer sulfonamide (*sul1*) and tetracycline (*tetA*) resistance was observed to be at 11.43% and 5.71% of isolates, respectively (Table 1). Overall, we observed low prevalence of resistance genes in concordance with the phenotypic antibiotic resistances detected by disk diffusion assays.

### Virulence Associated Phenotypes

**FIGURE 1:**
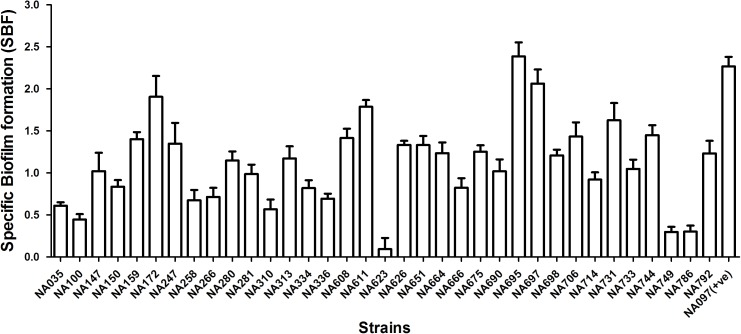
Biofilm formation in 35 *pks* positive *E. coli* isolates in M63 medium. Values are shown as mean of specific biofilm formation. Isolates demonstrating SBF values > 1.0 were considered as strong, 0.5–1.0 as moderate and <0.5 as weak biofilm formers. Majority of isolates (21/35) demonstrated strong biofilm formation; the remaining 10 and 4 isolates showed moderate and weak biofilm formation, respectively. NA097 was employed as a positive control in the biofilm formation assay.

**FIGURE 2:**
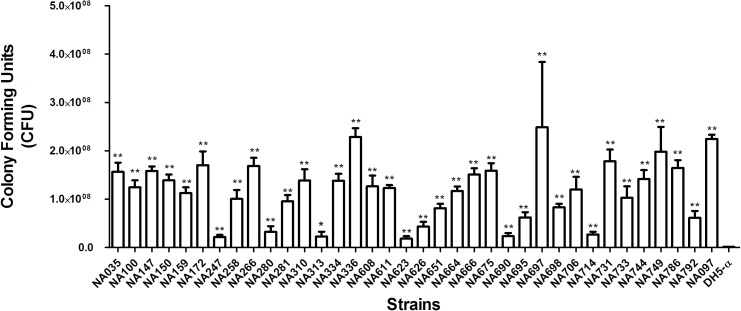
Serum resistance assay of *pks*-positive isolates in human serum. Mann–Whitney *U* test was carried out for calculating the significant differences. Significant differences were indicated by asterisks and *p* ≤ 0.05 was considered to be significant. ^∗^*p*-value ≤ 0.05, ^∗∗^*p*-value ≤ 0.01. NA097 was taken as the positive control, while DH5-α served as the negative control.

The isolates were analyzed for siderophore production, biofilm formation, and serum resistance which are essential ExPEC virulence properties. All *pks*-positive isolates formed orange halos on Chrome Azurol S plates, confirming the production of siderophores. Biofilm formation assay was performed twice in triplicates for all the *pks* positive isolates in order to determine their biofilm forming capabilities and was documented by specific biofilm formation (SBF) values. Isolates showing SBF values greater than 1 were designated as strong biofilm formers, 0.5–1.0 as moderate biofilm formers and those showing less than 0.5 were considered as weak biofilm formers (Figure 1). Majority (21/35) of the isolates tested were strong biofilm formers while 10/35 isolates moderately formed the biofilm and 4/35 isolates were weak biofilm formers. Bacterial resistance to the bactericidal activity of human serum was also assessed and the number of CFU were found to be significantly higher for all the *pks* positive isolates after 3 h of incubation in the human serum as compared to the negative control, indicative of the fact that all the isolates were resistant to human serum. Serum resistance was performed twice in technical triplicate(s) (Figure 2) and *p*-values were obtained using Mann–Whitney *U* test.

## Discussion

The *pks* island encodes enzymes that are able to synthesize colibactin, a genotoxin that could induce host DNA damage and its presence may contribute to increased virulence and severe disease outcomes. Ever since the description of colibactin by Nougayrède et al. (2006), many studies were undertaken to develop a comprehensive understanding of this bacterial genotoxin (Bossuet-Greif et al., 2018; Faïs et al., 2018). However, epidemiological data on the prevalence of the same and factors associated with *pks*-positive *E. coli* isolates, particularly from the Southern World have not been documented. The present study showed that 35 out of the 462 clinical ExPEC isolates were positive for all the four genes, indicating the presence of complete *pks* island(s) which might be able to synthesize functional colibactin. Thus, the overall prevalence was found to be 7.6% and this constitutes first epidemiological data on *pks* island harboring *E. coli* from India. In contrast, reports from other countries, such as those by Johnson et al. (2008) and Shimpoh et al. (2017) demonstrated high *pks* prevalence among clinical *E. coli*.

Our previous studies have suggested that majority of the *pks* negative clinical *E. coli* from India were genetically diverse and had a high prevalence of antibiotic resistance; these studies also identified and characterized the clonally evolving pandemic sequence type 131 *E. coli* isolates in India (Jadhav et al., 2011; Ranjan et al., 2016; Hussain et al., 2017; Shaik et al., 2017). However, the role of colibactin in the emergence of lineage specific virulence in *E. coli* that were comparatively less resistant to antibiotics has been shown herein for the first time from India. Such lineages could become a matter of public health concern and analysis of the underlying strains would be of great importance given the high burden of infectious diseases in this region. Thus, the findings of this study have implications for better understanding of the epidemiological context of pathogenic *E. coli* in human diseases.

*Escherichia coli* harboring *pks* island was reported to be strongly associated with bacteremia and human colorectal tumors (Johnson et al., 2008; Buc et al., 2013). Recent reports suggest that mice infected with colibactin positive *E. coli* had significantly lower survival rates compared to those infected with isogenic colibactin-negative mutant(s) (Marcq et al., 2014). It has been further demonstrated that *pks*-positive *E. coli* infection induces cellular senescence and concurrently produces growth factors which promote tumor growth (Secher et al., 2013; Cougnoux et al., 2014; Dalmasso et al., 2015). The *pks*-positive *E. coli* isolates in the present study were detected in different specimen types including urine, blood and pus. Therefore, it can be surmised that the *pks*-positive *E. coli* might contribute in many invasive and non-invasive infections at different anatomic sites.

Previous studies reported that the *pks* island was majorly detected in *E. coli* phylogenetic group B2 strains, which are mainly documented as extraintestinal pathogens (Taieb et al., 2016; Sarshar et al., 2017). Our results were in line with these observations as the *pks-*positive isolates in our study also belonged predominantly to phylogroup B2 (97%), except for one isolate which belonged to phylogroup D (3%) (Table 1). The pathogenic strains of *E. coli* mainly belong to group B2 and, to a lesser extent, group D and frequently harbor higher number of virulence-factors than group A and group B1 strains (Picard et al., 1999; Johnson et al., 2008).

Several studies have demonstrated a strong correlation between the presence of virulence genes and the pathogenic spectrum of *E. coli* strains (Bien et al., 2012). These include multiple ExPEC-associated virulence genes such as adhesins, invasins, secretory toxins, and iron scavenging systems (Johnson, 1991). Accordingly, the virulent nature of *pks*-positive *E. coli* isolates was supported by our findings as 100% of them were positive for *fimH* (D mannose specific adhesin of minor fimbrial component), *sfaD/E* (s-fimbrial adhesin) and *usp* (uropathogenic specific protein) while ≥ 30% of them were positive for *iucD* (enzyme for siderophore aerobactin synthesis), *sat* (secreted autotransporter vacuolating cytotoxin), *cvaC* (colicin-V precursor) and *ibeA* (invasion protein). The afimbrial adhesin gene, *afa* was found to be completely absent (Table 1). These findings could be attributed to the pathogenic potential of the *pks*-positive strains along with the presence of many other virulence determinants. Furthermore, in the present study, siderophore production was detected in all *pks*-positive isolates; this observation was consistent with previous reports on the positive correlation between the presence of *pks* island and iron scavenging systems among the B2 *E. coli* strains (Martin et al., 2017). The localization of *pks* island within the High-Pathogenicity Island (HPI) and its physical association with siderophore biosynthesis gene cluster has also been described in other members of *Enterobacteriaceae* (Putze et al., 2009). A majority of *pks*-positive *E. coli* isolates demonstrated high biofilm forming capabilities in M63 medium (Figure 1) and all the *pks*-positive isolates tested were found to be resistant to serum bactericidal activity (Figure 2). We speculate that these genotypic and phenotypic virulence traits expressed by B2 *pks*-positive *E. coli* could act as fitness factors in order to colonize and initiate/establish infection in intestinal and extra-intestinal sites.

Antibiotic susceptibility of the *pks*-positive isolates was performed and the isolates were observed to be uniquely associated with low antimicrobial resistance, this finding is consistent with the previous reports which have suggested that *pks* island harboring *E. coli* exhibit reduced antibiotic resistance (Chen et al., 2017; Sarshar et al., 2017). This observation could likely be due to opportunist *E. coli* infections arising from intestinal microbiota. Additionally, detailed characterization of such isolates from fecal and clinical samples is needed to certainly understand the reason behind such an observation. Multidrug resistance and ESBL production was observed in only 11.42 and 37% isolates, respectively (Table 1). Previous studies from our group have shown MDR rates of 95% for clinical ST131 strains, and 91% for clinical and stool non-ST131 strains (Hussain et al., 2014). We have also previously reported MDR rates of 100% for metallo-(β-lactamase (MBL) producing *E. coli* isolates (Ranjan et al., 2016) and 67% for isolates from skin and soft tissue infections (Ranjan et al., 2017). The *pks* positive isolates in contrast demonstrated lesser rates of antimicrobial resistance compared to the *pks* negative isolates characterized from the similar settings in our previous studies. All nine *bla*_CTXM-15_ positive *E. coli* isolates were observed to be ESBL producers (Table 1), although further screening for other groups of CTX-M genes, and other ESBL classes are warranted. In concordance with the antimicrobial susceptibility results, molecular detection of antimicrobial resistance genes showed a low prevalence of *sul1* (sulphonamide resistance), *tetA* (tetracycline resistance), *aac(6*^′^*)-Ib* (aminoglycoside resistance), *bla*_CTX-M-15_ (extended spectrum-β-lactamases) and *bla*_TEM-1_ (broad spectrum-β-lactamases) genes (Table 1).

## Conclusion

The prevalence of colibactin producing *E. coli* was found to be moderate among clinical *E. coli* isolates in our collection. These isolates harbored multiple virulence genes/traits and demonstrated relatively low antimicrobial resistance. These findings comprise essential baseline data required to understand aspects of functional molecular infection epidemiology of possibly genotoxic phenotypes of *E. coli* and their clinical significance. We hope to extend these studies at genomic and landscape scales to gain further insights into evolution, adaptation and dissemination of such isolates at clinical, community and ecosystem levels.

## Author Contributions

AS designed and performed all experiments with assistance from AR, SJ, AH, and SS. AS was responsible for all of the data and served as the guarantor for the manuscript. RB, MA, and LW participated in detailed discussions, advised on the interpretation of some of the results and, contributed to the writing and editing of the manuscript. NA conceived the study and provided overarching supervision, laboratory facilities and resources, interpreted and discussed results, and wrote/edited the draft and final versions of the manuscript. All authors contributed to the development of the manuscript and its display items.

## Conflict of Interest Statement

The authors declare that the research was conducted in the absence of any commercial or financial relationships that could be construed as a potential conflict of interest.

## References

[B1] Ahmed N. Dobrindt U. Hacker J. Hasnain S. E. (2008). Genomic fluidity and pathogenic bacteria: applications in diagnostics, epidemiology and intervention. *Nat. Rev. Microbiol.* 6 387–394. 10.1038/nrmicro1889 18392032

[B2] Avasthi T. S. Kumar N. Baddam R. Hussain A. Nandanwar N. Jadhav S. (2011). Genome of multidrug-resistant uropathogenic *Escherichia coli* strain NA114 from India. *J. Bacteriol.* 193 4272–4273. 10.1128/JB.05413-11 21685291 PMC3147708

[B3] Bien J. Sokolova O. Bozko P. (2012). Role of uropathogenic *escherichia coli* virulence factors in development of urinary tract infection and kidney damage. *Int. J. Nephrol.* 2012:681473. 10.1155/2012/681473 22506110 PMC3312279

[B4] Bossuet-Greif N. Vignard J. Taieb F. Mirey G. Dubois D. Petit C. (2018). The colibactin genotoxin generates DNA interstrand cross-links in infected cells. *mBio* 9:e02393-17. 10.1128/mBio.02393-17 29559578 PMC5874909

[B5] Buc E. Dubois D. Sauvanet P. Raisch J. Delmas J. Darfeuille-Michaud A. (2013). High prevalence of mucosa-associated *E. coli* producing cyclomodulin and genotoxin in colon cancer. *PLoS One* 8:e56964. 10.1371/journal.pone.0056964 23457644 PMC3572998

[B6] Chen Y.-T. Lai Y.-C. Tan M.-C. Hsieh L.-Y. Wang J.-T. Shiau Y.-R. (2017). Prevalence and characteristics of pks genotoxin gene cluster-positive clinical *Klebsiella pneumoniae* isolates in Taiwan. *Sci. Rep.* 7:43120. 10.1038/srep43120 28233784 PMC5324043

[B7] Clermont O. Christenson J. K. Denamur E. Gordon D. M. (2013). The Clermont *Escherichia coli* phylo-typing method revisited: improvement of specificity and detection of new phylo-groups. *Environ. Microbiol. Rep.* 5 58–65. 10.1111/1758-2229.12019 23757131

[B8] CLSI (2013). *Performance Standards for Antimicrobial Disk and Dilution Susceptibility Tests for Bacteria Isolated From Animals; Approved Standard. VET01-A4*, 4 Edn. Wayne, PA: Clin. Lab. Stand. Inst.

[B9] Cougnoux A. Dalmasso G. Martinez R. Buc E. Delmas J. Gibold L. (2014). Bacterial genotoxin colibactin promotes colon tumour growth by inducing a senescence-associated secretory phenotype. *Gut* 63 1932–1942. 10.1136/gutjnl-2013-305257 24658599

[B10] Croxen M. A. Finlay B. B. (2010). Molecular mechanisms of *Escherichia coli* pathogenicity. *Nat. Rev. Microbiol.* 8 26–38. 10.1038/nrmicro2265 19966814

[B11] Cuevas-Ramos G. Petit C. R. Marcq I. Boury M. Oswald E. Nougayrède J.-P. (2010). *Escherichia coli* induces DNA damage *in vivo* and triggers genomic instability in mammalian cells. *Proc. Natl. Acad. Sci. U.S.A.* 107 11537–11542. 10.1073/pnas.1001261107 20534522 PMC2895108

[B12] Dalmasso G. Cougnoux A. Delmas J. Darfeuille-Michaud A. Bonnet R. (2015). The bacterial genotoxin colibactin promotes colon tumor growth by modifying the tumor microenvironment. *Gut Microbes* 5 675–680. 10.4161/19490976.2014.969989 25483338 PMC4615906

[B13] Faïs T. Delmas J. Barnich N. Bonnet R. Dalmasso G. (2018). Colibactin: more than a new bacterial toxin. *Toxins* 10:151. 10.3390/toxins10040151 29642622 PMC5923317

[B14] Hussain A. Ewers C. Nandanwar N. Guenther S. Jadhav S. Wieler L. H. (2012). Multi-resistant uropathogenic *Escherichia coli* from an endemic zone of urinary tract infections in India: genotypic and phenotypic characteristics of ST131 isolates of the CTX-M-15 extended-spectrum-beta-lactamase producing lineage. *Antimicrob. Agents Chemother.* 56 6358–6365. 10.1128/AAC.01099-12 23045357 PMC3497203

[B15] Hussain A. Ranjan A. Nandanwar N. Babbar A. Jadhav S. Ahmed N. (2014). Genotypic and phenotypic profiles of *Escherichia coli* isolates belonging to clinical sequence type 131 (ST131), clinical non-ST131, and fecal non-ST131 lineages from India. *Antimicrob. Agents Chemother.* 58 7240–7249. 10.1128/AAC.03320-14 25246402 PMC4249578

[B16] Hussain A. Shaik S. Ranjan A. Nandanwar N. Tiwari S. K. Majid M. (2017). Risk of transmission of antimicrobial resistant *Escherichia coli* from commercial broiler and free-range retail chicken in India. *Front. Microbiol.* 8:2120. 10.3389/fmicb.2017.02120 29180984 PMC5694193

[B17] Jadhav S. Hussain A. Devi S. Kumar A. Parveen S. Gandham N. (2011). Virulence characteristics and genetic affinities of multiple drug resistant uropathogenic *Escherichia coli* from a semi urban locality in India. *PLoS One* 6:e18063. 10.1371/journal.pone.0018063 21464963 PMC3064663

[B18] Johnson J. R. (1991). Virulence factors in *Escherichia coli* urinary tract infection. *Clin. Microbiol. Rev.* 4 80–128. 10.1128/CMR.4.1.80.Updated 1672263 PMC358180

[B19] Johnson J. R. Johnston B. Kuskowski M. A. Nougayrede J.-P. Oswald E. (2008). Molecular epidemiology and phylogenetic distribution of the *Escherichia coli* pks genomic island. *J. Clin. Microbiol.* 46 3906–3911. 10.1128/JCM.00949-08 18945841 PMC2593299

[B20] Johnson J. R. Russo T. A. (2002). Extraintestinal pathogenic *Escherichia coli*: “The other bad *E coli*”. *J. Lab. Clin. Med.* 139 155–162. 10.1067/mlc.2002.121550 11944026

[B21] Kaper J. B. Nataro J. P. Mobley H. L. (2004). Pathogenic *Escherichia coli*. *Nat. Rev. Microbiol.* 2 123–140. 10.1038/nrmicro818 15040260

[B22] Marcq I. Martin P. Payros D. Cuevas-Ramos G. Boury M. Watrin C. (2014). The genotoxin colibactin exacerbates lymphopenia and decreases survival rate in mice infected with septicemic *Escherichia coli*. *J. Infect. Dis.* 210 285–294. 10.1093/infdis/jiu071 24489107

[B23] Martin P. Tronnet S. Garcie C. Oswald E. (2017). Interplay between siderophores and colibactin genotoxin in *Escherichia coli*. *IUBMB Life* 69 435–441. 10.1002/iub.1612 28295919

[B24] Messerer M. Fischer W. Schubert S. (2017). Investigation of horizontal gene transfer of pathogenicity islands in *Escherichia coli* using next-generation sequencing. *PLoS One* 12:e0179880. 10.1371/journal.pone.0179880 28732043 PMC5521745

[B25] Monstein H. J. Östholm-Balkhed,Å Nilsson Nilsson M. V. Dornbusch M. K. Nilsson L. E. (2007). Multiplex PCR amplification assay for the detection of blaSHV, blaTEM and blaCTX-M genes in *Enterobacteriaceae*. *APMIS* 115 1400–1408. 10.1111/j.1600-0463.2007.00722.x 18184411

[B26] Nandanwar N. Janssen T. Kühl M. Ahmed N. Ewers C. Wieler L. H. (2014). Extraintestinal pathogenic *Escherichia coli* (ExPEC) of human and avian origin belonging to sequence type complex 95 (STC95) portray indistinguishable virulence features. *Int. J. Med. Microbiol.* 304 835–842. 10.1016/j.ijmm.2014.06.009 25037925

[B27] Nougayrède J.-P. Homburg S. Taieb F. Boury M. Brzuszkiewicz E. Gottschalk G. (2006). *Escherichia coli* induces DNA double-strand breaks in eukaryotic cells. *Science* 313 848–851. 10.1126/science.1127059 16902142

[B28] Nougayrède J.-P. Taieb F. De Rycke J. Oswald E. (2005). Cyclomodulins: bacterial effectors that modulate the eukaryotic cell cycle. *Trends Microbiol.* 13 103–110. 10.1016/j.tim.2005.01.002 15737728

[B29] Picard B. Garcia J. S. Gouriou S. Duriez P. Brahimi N. Bingen E. (1999). The link between phylogeny and virulence in *Escherichia coli* extraintestinal infection? *Infect. Immun.* 67 546–553. 9916057 10.1128/iai.67.2.546-553.1999 PMC96353

[B30] Putze J. Hennequin C. Nougayrède J. P. Zhang W. Homburg S. Karch H. (2009). Genetic structure and distribution of the colibactin genomic island among members of the family Enterobacteriaceae. *Infect. Immun.* 77 4696–4703. 10.1128/IAI.00522-09 19720753 PMC2772509

[B31] Qumar S. Majid M. Kumar N. Tiwari S. K. Semmler T. Devi S. (2017). Genome dynamics and molecular infection epidemiology of multidrug-resistant *Helicobacter* pullorum isolates obtained from broiler and free-range chickens in India. *Appl. Environ. Microbiol.* 83:e02305-16. 10.1128/AEM.02305-16 27815276 PMC5165125

[B32] Ranjan A. Shaik S. Mondal A. Nandanwar N. Hussain A. Semmler T. (2016). Molecular epidemiology and genome dynamics of New Delhi Metallo-β-Lactamase-producing extraintestinal pathogenic *Escherichia coli* strains from India. *Antimicrob. Agents Chemother.* 60 6795–6805. 10.1128/AAC.01345-16 27600040 PMC5075088

[B33] Ranjan A. Shaik S. Nandanwar N. Hussain A. Tiwari S. K. Semmler T. (2017). Comparative genomics of *Escherichia coli* isolated from skin and soft tissue and other extraintestinal infections. *mBio* 8:e01070-17. 10.1128/mBio.01070-17 28811343 PMC5559633

[B34] Sarshar M. Scribano D. Marazzato M. Ambrosi C. Aprea M. R. Aleandri M. (2017). Genetic diversity, phylogroup distribution and virulence gene profile of pks positive *Escherichia coli* colonizing human intestinal polyps. *Microb. Pathog.* 112 274–278. 10.1016/j.micpath.2017.10.009 28987619

[B35] Schwyn B. Neilands J. B. (1987). Universal chemical assay for the detection and determination of siderophores. *Anal. Biochem.* 160 47–56. 10.1016/0003-2697(87)90612-9 2952030

[B36] Secher T. Samba-Louaka A. Oswald E. Nougayrède J. P. (2013). *Escherichia coli* producing colibactin triggers premature and transmissible senescence in mammalian cells. *PLoS One* 8:e77157. 10.1371/journal.pone.0077157 24116215 PMC3792898

[B37] Shaik S. Ranjan A. Tiwari S. K. Hussain A. Nandanwar N. Kumar N. (2017). Comparative genomic analysis of globally dominant ST131 clone with other epidemiologically successful extraintestinal pathogenic *Escherichia coli* (ExPEC) lineages. *mBio* 8:e01596-17. 10.1128/mBio.01596-17 29066550 PMC5654935

[B38] Shimpoh T. Hirata Y. Ihara S. Suzuki N. Kinoshita H. Hayakawa Y. (2017). Prevalence of *pks*-positive *Escherichia coli* in Japanese patients with or without colorectal cancer. *Gut Pathog.* 9:35. 10.1186/s13099-017-0185-x 28616082 PMC5468999

[B39] Smith J. L. Fratamico P. M. Gunther N. W. (2007). Extraintestinal pathogenic *Escherichia coli*. *Foodborne Pathog. Dis.* 4 134–163. 10.1089/fpd.2007.0087 17600482

[B40] Taieb F. Petit C. Nougayrède J.-P. Oswald E. (2016). The Enterobacterial genotoxins: cytolethal distending toxin and colibactin. *EcoSal Plus.* 10.1128/ecosalplus.ESP-0008-2016 27419387 PMC11575708

