# Supplementary material for: Molecular Genetic and Functional Analysis of pks-Harboring, Extra-Intestinal Pathogenic Escherichia coli From India
Source: Front Microbiol. 2018 Nov 15;9:2631. doi: 10.3389/fmicb.2018.02631 (PMC6249908; doi:10.3389/fmicb.2018.02631)
Supplement: Supplementary file 1 [file Table_1.DOCX]

| **Strain** | **Gender** | **Age** | **Disease status** | **Source** |
| --- | --- | --- | --- | --- |
| NA035 | Male | 65 | Septicaemia | Urine |
| NA100 | Female | 45 | Septicaemia | Urine |
| NA147 | Female | 34 | UTI | Urine |
| NA150 | Female | 19 | Pyelonephritis | Urine |
| NA159 | Female | 22 | Cystitis | Urine |
| NA172 | Male | 60 | Prostitis | Urine |
| NA247 | Male | 20 | Cystitis | Urine |
| NA258 | Male | 55 | UTI | Urine |
| NA266 | Female | 20 | UTI | Urine |
| NA280 | Male | 35 | Septicaemia | Urine |
| NA281 | Female | 20 | Cystitis | Urine |
| NA310 | Female | 26 | UTI | Urine |
| NA313 | Female | 17 | UTI | Urine |
| NA334 | Female | 26 | UTI | Urine |
| NA336 | Female | 30 | UTI | Urine |
| NA608 | Male | 2 | Chronic UTI | Urine |
| NA611 | Male | 2 | Chronic UTI and severe abdominal Pain | Blood |
| NA623 | Male | 75 | UTI | Urine |
| NA626 | Female | 8 | Renal calculi | Urine |
| NA651 | Female | 55 | Surgical site infection | Pus |
| NA664 | Male | 36 | UTI | Urine |
| NA666 | Female | 28 | Unknown | Urine |
| NA675 | Male | 60 | UTI | Urine |
| NA690 | Female | 32 | Unknown | Urine |
| NA695 | Female | 30 | Unknown | Urine |
| NA697 | Female | 23 | Unknown | Urine |
| NA698 | Female | 39 | Unknown | Urine |
| NA706 | Male | 50 | Unknown | Pus |
| NA714 | Male | 11 | UTI | Urine |
| NA731 | Female | 35 | UTI | Urine |
| NA733 | Female | 22 | Renal calculi | Urine |
| NA744 | Female | 35 | Haematuria | Urine |
| NA749 | Female | 20 | Unknown | Urine |
| NA786 | Female | 40 | Omphitis | Pus |
| NA792 | Male | 60 | Diabetic Foot | Pus |

**Supplementary Table S1:** Clinical details of the *pks* positive isolates
